# Targeting Ferroptosis to Overcome Radioresistance and Enhance Immunotherapy in Colorectal Cancer

**DOI:** 10.3390/cells15110993

**Published:** 2026-05-28

**Authors:** Sara Soltani Tehrani, Samuel Isaac Olson, Karishma Kundu, Sylvain Ferrandon, Matthew F. Kalady

**Affiliations:** 1 The Ohio State University James Comprehensive Cancer Center, College of Medicine, The Ohio State University, Columbus, OH 43210, USA; sara.soltanitehrani@osumc.edu (S.S.T.); samuel.olson@osumc.edu (S.I.O.); karishmakundu95@gmail.com (K.K.); sylvain.ferrandon@osumc.edu (S.F.); 2 Division of Colon and Rectal Surgery, Department of Surgery, The Ohio State University College of Medicine, Columbus, OH 43210, USA

**Keywords:** rectal cancer, radiotherapy, ferroptosis, redox metabolism, radioresistance, biomarkers, immunogenic cell death, total neoadjuvant therapy

## Abstract

**Highlights:**

**What are the main findings?**

- Ferroptosis links radiation-induced oxidative stress, chemotherapy response, and antitumor immunity in locally advanced rectal cancer.
- The immune effects of ferroptosis are context-dependent, with tumor-cell ferroptosis potentially enhancing immunity and non-selective lipid peroxidation suppressing dendritic-cell and CD8^+^ T-cell function.

**What are the implications of the main findings?**

- Ferroptosis-related biomarkers may improve response prediction in MSS/pMMR rectal cancer, especially in patients considered for watch-and-wait management.
- Tumor-selective ferroptosis may provide a rational strategy to enhance radiotherapy, total neoadjuvant therapy, and immune checkpoint blockade without compromising antitumor immunity.

**Abstract:**

Locally advanced rectal cancer is commonly treated using total neoadjuvant therapy (TNT), which integrates radiotherapy with systemic chemotherapy to improve tumor downstaging, local control, and long-term oncologic outcomes. Despite its central role in treatment, responses to radiotherapy remain highly heterogeneous. While some tumors undergo complete regression, others exhibit intrinsic or acquired treatment resistance, resulting in incomplete tumor control while experiencing treatment-related toxicity. Understanding the biological determinants that govern radiation sensitivity in rectal cancer, therefore, represents a major clinical challenge. Ionizing radiation induces tumor cell death primarily through the generation of reactive oxygen species (ROS) and DNA damage, particularly DNA double-strand breaks. In addition to nuclear DNA injury, radiation-induced oxidative stress can initiate lipid peroxidation within cellular membranes. When lipid peroxide accumulation exceeds the capacity of cellular antioxidant systems, this process can trigger ferroptosis, an iron-dependent form of regulated cell death driven by phospholipid oxidation. Ferroptotic susceptibility is regulated by interconnected metabolic pathways, including cystine transport through system Xc^−^ (SLC7A11/SLC3A2), glutathione synthesis, glutathione peroxidase-4 (GPX4) activity, iron metabolism, and membrane lipid remodeling. Recent evidence further indicates that ferroptosis intersects with antitumor immunity. Ferroptotic tumor cells release oxidized lipid mediators and damage-associated molecular signals that can influence immune activation, while interferon-γ produced by activated CD8^+^ T cells during immune checkpoint blockade suppresses SLC7A11 expression, limiting cystine uptake and promoting ferroptotic tumor cell death. These findings suggest that ferroptosis represents a mechanistic interface between tumor metabolic vulnerability and immune-mediated cytotoxicity. This interaction is particularly relevant in colorectal cancer biology, where immune checkpoint inhibitors demonstrate clinical benefit primarily in tumors with deficient mismatch repair or microsatellite instability-high (MSI-H) status. The vast majority of rectal cancers are microsatellite stable (MSS) and exhibit limited responsiveness to immunotherapy due to reduced immunogenicity and immune exclusion within the tumor microenvironment. Strategies capable of increasing tumor immunogenicity in this setting are therefore of considerable interest. In this review, we examine the molecular mechanisms linking radiation-induced oxidative stress to ferroptosis and tumor immunity in colorectal cancer, while focusing on the clinical context of radiotherapy in rectal cancer. We discuss how lipid metabolism, iron homeostasis, cysteine-dependent antioxidant systems, and immune signaling pathways converge to regulate ferroptotic vulnerability and radiation response. We further explore the therapeutic potential of integrating radiotherapy, ferroptosis-targeting strategies, and immunotherapy to overcome radioresistance and improve treatment outcomes in colorectal cancer.

## 1. Introduction

Colorectal cancer (CRC) is the third most commonly diagnosed cancer and the second leading cause of cancer-related mortality in the United States [1]. Despite advances in prevention, screening, and treatment, the incidence of CRC is increasing among individuals younger than 50 years, highlighting an emerging public health concern [1,2,3]. Within this group, rectal cancer represents a biologically and anatomically distinct disease that requires specialized therapeutic strategies. For patients with locally advanced rectal cancer, treatment commonly includes total neoadjuvant therapy (TNT), a multimodal approach integrating radiotherapy with systemic chemotherapy prior to surgical resection [4]. This strategy aims to improve tumor downstaging, reduce local recurrence, and increase the likelihood of organ preservation [5] in selected patients [4,6,7]. While some tumors undergo marked regression or even complete clinical and pathologic response, others show only limited regression or persistent resistant disease despite receiving similar treatment regimens [8,9]. This heterogeneity in treatment response indicates that radiation sensitivity is multifactorial, including response to DNA damage, tumor metabolic state, antioxidant capacity, and interactions with the immune microenvironment [10]. Among these mechanisms, ferroptosis has emerged as a particularly relevant process. Ferroptosis is an iron-dependent form of regulated cell death driven by the accumulation of oxidized phospholipids in cellular membranes [11]. Because ionizing radiation generates oxidative stress and promotes conditions favorable to lipid peroxidation, ferroptosis may represent an important component of radiation-induced tumor death beyond canonical DNA damage responses [12,13]. At the same time, ferroptosis is increasingly recognized as functionally connected to antitumor immunity [14]. Oxidative lipid damage, inflammatory mediators, and metabolic vulnerabilities within the tumor microenvironment can influence both tumor-cell ferroptotic susceptibility and immune-cell activity [15,16]. This relationship is especially relevant in colorectal cancer, where most tumors are microsatellite stable and remain poorly responsive to immune checkpoint blockade, creating interest in biological strategies that could enhance tumor immunogenicity and therapeutic response [17,18]. In this review, we examine how radiation-induced oxidative stress, ferroptosis-regulatory pathways, and immune signaling, with particular emphasis on the role of these processes in rectal cancer radiation response [19]. We discuss the molecular basis of ferroptosis, its interaction with tumor immunity, the role of innate immune signaling pathways such as cGAS–STING, and the therapeutic potentials to overcome radioresistance.

## 2. Radiotherapy and Ferroptosis in Rectal Cancer

### 2.1. Radiation-Induced Oxidative Stress Beyond DNA Damage

Radiotherapy remains a central component of treatment for locally advanced rectal cancer, particularly within total neoadjuvant therapy regimens [11]. Its cytotoxic activity has traditionally been explained through the induction of DNA damage, especially DNA double-strand breaks, which activate DNA damage response pathways and can ultimately lead to cell-cycle arrest or cell death [20]. However, this DNA-centered model does not fully explain the heterogeneity of radiation response observed in rectal cancer [15,16]. Some tumors undergo marked regression or complete clinical response, whereas others persist despite receiving similar radiation and chemotherapy backbones. This variation suggests that radiation response is shaped not only by DNA repair capacity, but also by the oxidative and metabolic state of the tumor. Ionizing radiation generates reactive oxygen species through radiolysis of intracellular water and through secondary mitochondrial stress [21]. These reactive species damage proteins, nucleic acids, and membrane lipids [20,21]. Polyunsaturated fatty acid-containing phospholipids are particularly vulnerable because they can undergo chain lipid peroxidation [11,22,23,24]. When lipid peroxide formation exceeds the capacity of antioxidant systems to detoxify these products, cells may enter ferroptosis, an iron-dependent form of regulated cell death driven by lethal membrane lipid oxidation. In this context, the most clinically relevant question for rectal cancer is not simply whether ferroptosis exists, but whether radiotherapy creates a targetable interval in which lipid-peroxidation stress overwhelms tumor antioxidant defenses [23,25].

**Figure 1:**
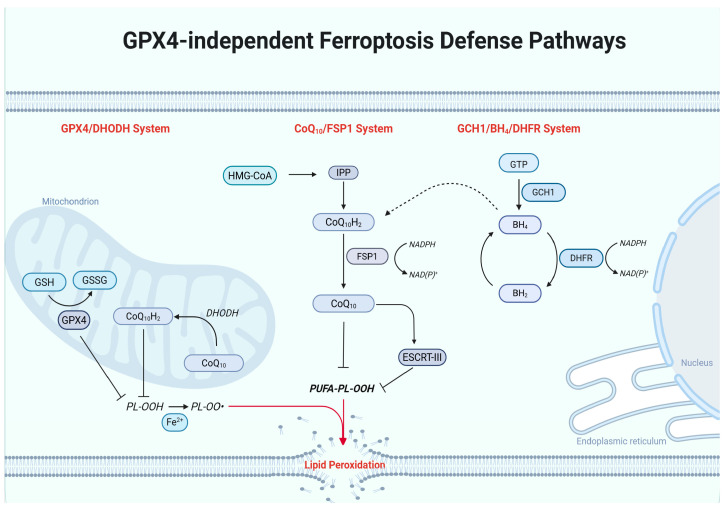
GPX4-independent ferroptosis defense pathways. Cells suppress lipid peroxidation through parallel GPX4-independent antioxidant systems. The FSP1–CoQ10 axis reduces lipid radicals at cellular membranes, the DHODH pathway maintains mitochondrial CoQ redox balance, and the GCH1–BH4–DHFR pathway generates radical-trapping antioxidants that protect polyunsaturated phospholipids from peroxidation, collectively limiting ferroptotic cell death. Abbreviations: BH2, dihydrobiopterin; BH4, tetrahydrobiopterin; CoQ10, coenzyme Q10 (ubiquinone); CoQ10H_2_, ubiquinol; DHFR, dihydrofolate reductase; DHODH, dihydroorotate dehydrogenase; ESCRT-III, endosomal sorting complexes required for transport-III; FSP1, ferroptosis suppressor protein-1 (AIFM2); GCH1, GTP cyclohydrolase-1; GPX4, glutathione peroxidase-4; GSH, reduced glutathione; GSSG, oxidized glutathione; GTP, guanosine triphosphate; HMG-CoA, 3-hydroxy-3-methylglutaryl-coenzyme A; IPP, isopentenyl pyrophosphate; NAD(P)H, reduced nicotinamide adenine dinucleotide (phosphate); PL-OOH, phospholipid hydroperoxide; PL-OO, phospholipid peroxyl radical; PUFA-PL-OOH, polyunsaturated fatty acid-containing phospholipid hydroperoxide. (@BioRender).

The central protective mechanism against ferroptosis is the system Xc^−^ cystine transporter, which is composed of the transmembrane proteins SLC7A11 and SLC3A2 [26]. This antiporter imports extracellular cystine in exchange for intracellular glutamate. Once inside the cell, cystine is rapidly reduced to cysteine, which serves as the rate-limiting substrate for the synthesis of glutathione, the major intracellular antioxidant [27,28]. Glutathione (GSH) functions as a critical cofactor for Glutathione Peroxide 4 GPX4, a selenoenzyme that directly reduces phospholipid hydroperoxides (PL-OOH) to non-toxic lipid alcohols (PL-OH) [29]. GPX4 therefore acts as the principal enzymatic defense preventing the accumulation of lethal lipid peroxides within cellular membranes [30,31]. When the System Xc^−^-GSH-GPX4 axis is disrupted, lipid hydroperoxides accumulate and initiate ferroptosis [27,32]. Several metabolic factors regulate this pathway. High expression of SLC7A11 enhances cystine uptake and increases intracellular GSH levels, strengthening antioxidant capacity and suppressing ferroptosis [26]. Conversely, inhibition of system Xc^−^ or depletion of GSH compromises GPX4 activity and sensitizes cells to lipid peroxide accumulation. Additional ferroptosis defense pathways, including FSP1–CoQ10, DHODH-mediated mitochondrial CoQ protection, and the GCH1–BH4 axis, provide parallel antioxidant buffering [33,34] shown in (Figure 1). These mechanisms are biologically important, but in rectal cancer, the key translational issue is whether radiation and concurrent systemic therapy push tumor cells beyond these protective thresholds [35,36]. As many tumors, including colorectal cancer, upregulate SLC7A11 and GSH metabolism to tolerate oxidative stress, this pathway represents a critical determinant of resistance to ferroptotic death [12,27,37].

### 2.2. Radiotherapy as a Promoter of Ferroptotic Cell Death

Radiotherapy creates multiple biochemical conditions that favor ferroptosis [16,24]. First, radiation-induced ROS directly initiate lipid peroxidation in PUFA-containing membranes [15,38]. Second, radiation-induced metabolic stress can impair antioxidant defense systems, reducing the capacity of tumor cells to detoxify lipid hydroperoxides [15]. Third, radiation can alter intracellular iron homeostasis through mitochondrial dysfunction and oxidative damage to iron-storage proteins, thereby expanding the labile iron pool [15,39]. Together, these processes shift the balance between lipid peroxide generation and detoxification toward oxidative membrane damage. Importantly, the biochemical conditions that promote ferroptosis during radiotherapy also intersect with immune signaling pathways within the tumor microenvironment [15]. Oxidative stress, lipid peroxidation, and metabolic vulnerability can influence immune-mediated tumor killing, suggesting that ferroptosis may represent a mechanistic interface between radiation biology and antitumor immunity.

### 2.3. Iron Metabolism and Lipid Remodeling in Ferroptotic Vulnerability

**Figure 2:**
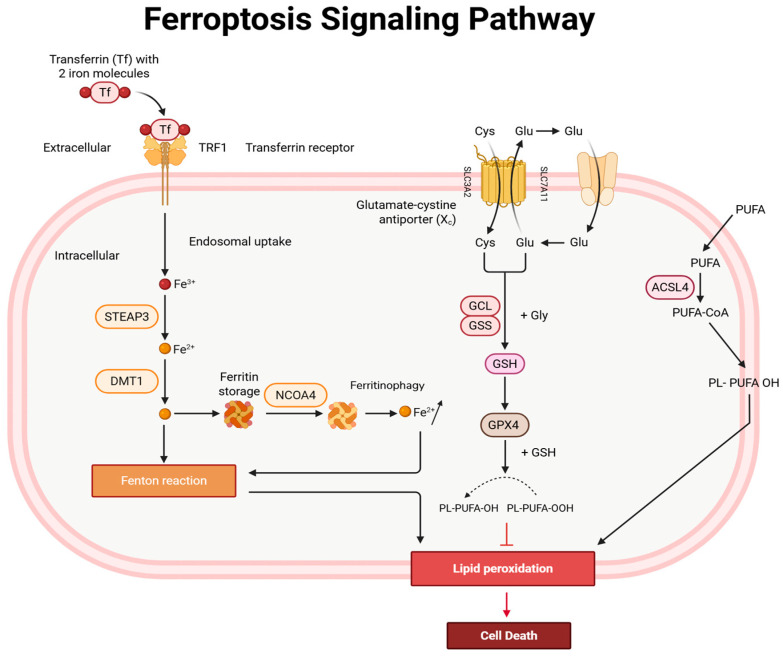
Ferroptosis signaling pathway. Schematic representation of the molecular pathway involved in ferroptosis. Abbreviations: ACSL4, acyl-CoA synthetase long-chain family member 4; Cys, cysteine; DMT1, divalent metal transporter 1; Fe^2+^, ferrous iron; Fe^3+^, ferric iron; GCL, glutamate–cysteine ligase; Glu, glutamate; GPX4, glutathione peroxidase 4; GSH, glutathione; GSS, glutathione synthetase; NCOA4, nuclear receptor coactivator 4; PL-PUFA-OH, phospholipid-bound polyunsaturated fatty acid alcohol; PL-PUFA-OOH, phospholipid-bound polyunsaturated fatty acid hydroperoxide; PUFA, polyunsaturated fatty acid; STEAP3, six-transmembrane epithelial antigen of prostate 3; Tf, transferrin. (@BioRender).

A defining feature of ferroptosis is its dependence on intracellular iron. Iron catalyzes the Fenton reaction, in which ferrous iron (Fe^2+^) converts hydrogen peroxide into highly reactive hydroxyl radicals that accelerate lipid peroxidation [11]. Increased intracellular iron, therefore, amplifies oxidative damage and promotes ferroptotic cell death [11,12,40]. Iron availability within cells is regulated through coordinated control of iron import, storage, and release [41]. Transferrin receptor 1 (TFR1) mediates uptake and increases intracellular iron pools, while ferritin stores excess iron in a non-reactive form [42]. Degradation of ferritin by autophagy (ferritinophagy) can release free iron, expand the labile iron pool, and promote ROS-mediated lipid oxidation [43,44]. The cargo receptor nuclear receptor coactivator-4 (NCOA4) mediates ferritinophagy by targeting ferritin complexes for lysosomal degradation [45]. Activation of this pathway increases intracellular iron availability and promotes lipid oxidation, thereby enhancing susceptibility to ferroptosis [46] (Figure 1). In addition to iron availability, membrane lipid composition strongly influences ferroptotic susceptibility. PUFAs such as arachidonic acid and adrenic acid are preferentially incorporated into phosphatidylethanolamine phospholipids through the action of enzymes, including acyl-CoA synthetase long-chain family member 4 (ACSL4) [40,42,47]. These PUFA-containing phospholipids serve as the primary substrates for lipid peroxidation during ferroptosis [40]. Cells enriched in PUFA-containing phospholipids, therefore, exhibit increased vulnerability to ferroptotic death because these specific lipids are highly susceptible to iron-dependent lipid peroxidation. When these membranes become overloaded with lipid peroxides (specifically PUFA-phosphatidylethanolamine), the integrity of the cell membrane is compromised, leading to rapid cell death [48,49] (Figure 2).

## 3. Radiotherapy, Total Neoadjuvant Therapy, and Ferroptotic Vulnerability in Rectal Cancer

### 3.1. Rectal Cancer Radiotherapy as a Context for Ferroptosis

In rectal cancer, radiotherapy is rarely delivered in isolation [50]. Commonly, there are two options within neoadjuvant treatment regimes. The first is long-course radiotherapy (LCRT), which typically delivers 45–50 Gy in daily fractions of 1.8–2 Gy over approximately 5 weeks and is commonly given with concurrent fluoropyrimidine-based chemotherapy such as 5-FU or capecitabine. Total neoadjuvant therapy (TNT) includes the addition of neoadjuvant independent systemic chemotherapy, such as FOLFOX, CAPOX, or modified FOLFIRINOX. The second radiotherapy option is short-course radiotherapy (SCRT), which consists of 25 Gy delivered in five 5-Gy fractions over one week [51,52]. SCRT can also be combined with separate systemic chemotherapy options as a TNT regimen [53]. The OPRA trial tested a randomized TNT approach with either induction chemotherapy followed by chemoradiotherapy or chemoradiotherapy followed by consolidation chemotherapy, and patients with a complete or near-complete response were offered a non-operative watch-and-wait management [54,55]. In the long-term OPRA update, organ preservation remained achievable in a substantial proportion of patients, but local regrowth occurred mainly within the first two to three years, emphasizing the need for better biomarkers of durable response. These agents may influence ferroptotic vulnerability by altering nucleotide stress, mitochondrial metabolism, iron handling, antioxidant capacity, and lipid peroxide detoxification. Therefore, ferroptosis in rectal cancer should be considered in the context of combined modality therapy rather than radiation alone.

For fluoropyrimidines, emerging CRC evidence suggests that ferroptosis can influence 5-FU response. Several studies have linked 5-FU resistance to enhanced antioxidant buffering and suppression of ferroptotic death. For example, PYCR1 and FAM98A have been implicated in limiting 5-FU-induced ferroptosis in CRC models, while 5-FU-resistant cells may acquire mitochondrial adaptations that create distinct ferroptosis vulnerabilities. These findings suggest that fluoropyrimidines may shift the ferroptotic threshold, although direct evidence in rectal chemoradiation remains limited. For capecitabine, which is commonly used during long-course chemoradiotherapy, the evidence remains even more limited and comes mainly from CRC cell-line studies rather than rectal cancer-specific models.

Oxaliplatin has stronger mechanistic links to ferroptosis in CRC. Suppression of the KIF20A/NUAK1/Nrf2/GPX4 signaling axis has been shown to induce ferroptosis and enhance oxaliplatin sensitivity in CRC [56]. In addition, Fusobacterium nucleatum has been reported to promote oxaliplatin resistance by inhibiting ferroptosis through GPX4-associated signaling. These data are clinically relevant because oxaliplatin-containing regimens are central to TNT [57,58]. They also suggest that ferroptosis resistance may contribute not only to radiotherapy resistance but also to chemotherapy resistance within the same treatment course.

The relationship between irinotecan or modified FOLFIRINOX and ferroptosis is less clearly defined [59]. Modified FOLFIRINOX is clinically important in TNT for high-risk locally advanced rectal cancer, but direct evidence showing how irinotecan-containing therapy alters ferroptotic threshold in rectal tumors is still sparse [60]. At present, it is reasonable to state that FOLFIRINOX may plausibly affect ferroptosis through DNA damage, mitochondrial stress, and redox remodeling, but this remains an inference rather than a demonstrated rectal cancer mechanism. This limitation should be acknowledged clearly to avoid overstating the evidence.

### 3.2. Short-Course Versus Long-Course Radiotherapy: Not Biologically Interchangeable

Long-course chemoradiotherapy delivers conventionally fractionated radiation with concurrent fluoropyrimidine therapy, typically over several weeks [61]. Short-course radiotherapy delivers larger radiation fractions over a shorter period and is often followed by systemic chemotherapy in TNT regimens [61]. These schedules differ not only in dose and timing but also in their effects on systemic immunity, lymphocyte depletion, and innate immune activation. Because of this, short-course and long-course radiotherapy should not be treated as biologically equivalent when discussing ferroptosis and immune response in rectal cancer [62].

Recent rectal adenocarcinoma studies suggest that short-course radiotherapy may produce a different immune profile than long-course treatment. Koukourakis and colleagues reported that short-course radiotherapy was associated with increased post-treatment IFNβ levels and less severe lymphopenia compared with long-course radiotherapy [63]. Elevated post-treatment IFNβ was also associated with improved pathological tumor regression. A more recent translational study further suggested that short-course radiotherapy may be less lymphodepleting and more likely to increase intratumoral T-cell infiltration than long-course radiotherapy [63]. These findings matter for ferroptosis because immune-mediated ferroptotic pressure depends, at least in part, on preserved effector immune function.

This distinction is also clinically relevant for organ preservation. In watch-and-wait strategies, treatment response is assessed after completion of neoadjuvant therapy using clinical examination, endoscopy, and MRI. In the OPRA trial, restaging occurred approximately 8 ± 4 weeks after TNT, and patients with complete or near-complete clinical response could enter watch-and-wait surveillance [64]. Because tumor regression can deepen over time, a single early post-radiotherapy biopsy may not capture the full biology of response. Ferroptosis-linked biomarker studies should therefore consider longitudinal sampling, including pretreatment tissue, early on-treatment or post-radiotherapy specimens when feasible, and post-TNT restaging samples aligned with organ-preservation decisions.

### 3.3. Candidate Ferroptosis Biomarkers for Rectal Cancer Response

A rectal-cancer-focused ferroptosis biomarker strategy should prioritize pathways with the strongest connection to radiation response and treatment resistance [65]. Based on current evidence, the most clinically relevant candidates include SLC7A11, GPX4, GSH-related metabolism, iron-handling proteins, lipid peroxidation markers, and regulators of antioxidant transcriptional programs such as NRF2. High SLC7A11 or GPX4 activity may indicate strong ferroptosis defense and relative resistance to radiation-induced lipid peroxidation [66]. Conversely, increased lipid peroxide accumulation, labile iron availability, or reduced antioxidant buffering may identify tumors more likely to undergo ferroptotic death after radiotherapy [67].

Importantly, these biomarkers should not be interpreted in isolation. Their clinical value will likely depend on treatment context, including whether the patient receives long-course chemoradiotherapy, short-course radiotherapy, consolidation chemotherapy, induction chemotherapy, or modified FOLFIRINOX-based TNT [68,69]. Biomarker timing is also essential. Pretreatment markers may predict baseline ferroptotic vulnerability, whereas post-treatment markers may reflect therapy-induced adaptation or residual resistant disease. For watch-and-wait decisions, the most useful biomarkers would be those that complement MRI, endoscopy, digital rectal examination, and circulating tumor DNA kinetics to distinguish tumors undergoing durable regression from those likely to regrow [69,70,71].

Overall, radiotherapy creates a biologically plausible ferroptosis window in rectal cancer, but the clinical translation of this concept remains early [16]. Current evidence supports ferroptosis as a promising mechanism of radiosensitization and immune modulation, yet rectal cancer-specific validation is still needed [72]. Future studies should test ferroptosis markers prospectively in clinically annotated rectal cancer cohorts, ideally with paired pretreatment and post-treatment samples, defined radiotherapy schedules, and standardized response endpoints including pathologic complete response, clinical complete response, organ preservation, and local regrowth [73].

## 4. Ferroptosis, Immunity, and MSS/pMMR Rectal Cancer

### 4.1. Why MSS/pMMR Rectal Cancer Is the Key Translational Setting

Most colorectal cancers are immunologically “cold,” particularly the microsatellite-stable (MSS) subtype, which accounts for approximately 80–85% of cases and is characterized by low tumor mutational burden, limited CD8^+^ T-cell infiltration, and a strongly immunosuppressive tumor microenvironment [74]. These features contribute to the poor clinical activity of immune checkpoint inhibitors in MSS CRC compared with MSI-H/dMMR tumors [75,76]. Ferroptosis may add another layer by increasing lipid-peroxidation-driven tumor-cell death and danger signaling. Radiotherapy can increase oxidative injury and antigen release, chemotherapy can reshape tumor redox state, and immune checkpoint blockade can increase CD8^+^ T-cell activity [76]. If tumor cells are pushed toward lipid peroxide overload while effector T cells are preserved, ferroptosis could enhance tumor killing and increase immune visibility [77]. However, the immune consequences of ferroptosis are context-dependent. Tumor-cell ferroptosis may support immune activation, but lipid peroxidation in dendritic cells or T cells may suppress antitumor immunity. Therefore, in MSS/pMMR rectal cancer, the therapeutic goal should be selective induction of ferroptosis in tumor cells while preserving dendritic-cell antigen presentation and CD8^+^ T-cell effector function [74]. The overall effect depends on which cells undergo ferroptosis, when it occurs, and to what extent it is induced [78].

Several foundational immune-ferroptosis studies come from non-rectal models and should be framed accordingly. Wang and colleagues showed that immunotherapy-activated CD8^+^ T cells can promote tumor-cell ferroptosis through IFNγ-mediated suppression of SLC7A11 and SLC3A2. Liao and colleagues demonstrated that ACSL4-dependent lipid remodeling can shape ferroptosis and antitumor immunity [14]. Ping and colleagues showed that PD-1 signaling can promote ferroptosis-related dysfunction in intratumoral CD8^+^ T cells through altered phospholipid metabolism [14]. These studies are highly informative, but they should be presented as conceptual evidence rather than direct rectal cancer evidence. The rectal cancer-specific question is how these mechanisms operate in MSS/pMMR tumors treated with radiotherapy, fluoropyrimidines, oxaliplatin-based chemotherapy, and emerging immunotherapy combinations.

### 4.2. CD8^+^ T Cells Promote Tumor Ferroptosis Through IFN-γ Signaling

**Figure 3:**
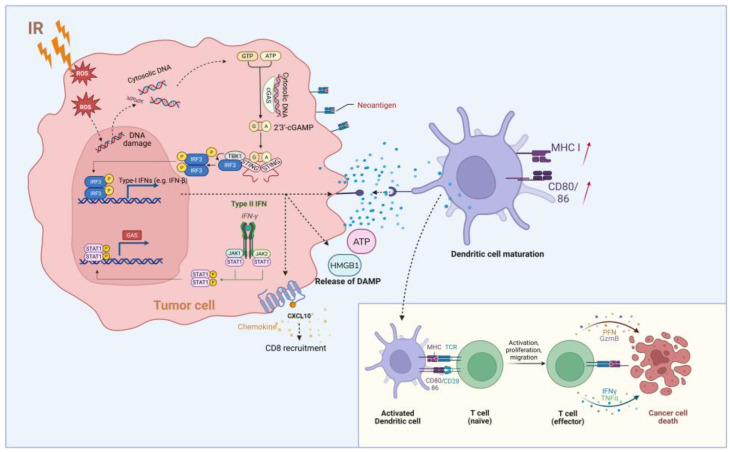
Radiotherapy-induced cGAS–STING signaling enhances dendritic-cell activation and antitumor T-cell responses. Abbreviations: IR, ionizing radiation; ROS, reactive oxygen species; dsDNA, double-stranded DNA; cGAS, cyclic GMP–AMP synthase; 2′3′-cGAMP, 2′3′-cyclic GMP–AMP; STING, stimulator of interferon genes; TBK1, TANK-binding kinase 1; IRF3, interferon regulatory factor 3; IFNβ, interferon-beta; JAK, Janus kinase; STAT, signal transducer and activator of transcription; MHC I, major histocompatibility complex class I; CD80/86, cluster of differentiation 80/86; PFN, perforin; GZMB, granzyme B; TNFα, tumor necrosis factor-alpha; IFNγ, interferon-gamma; ATP, adenosine triphosphate; GTP, guanosine triphosphate; TCR, T-cell receptor. (@BioRender).

Recent studies have shown that activated CD8^+^ T cells can promote tumor ferroptosis through IFN-γ signaling [78]. Upon binding to the IFN-γ receptor on tumor cells, IFN-γ activates the receptor-associated kinases JAK1 and JAK2, resulting in phosphorylation and nuclear translocation of STAT1 [79]. Activated STAT1 then suppresses the expression of the system x_c^−^ subunits SLC7A11 and SLC3A2, thereby limiting cystine uptake, depleting intracellular glutathione, and weakening GPX4-dependent detoxification of lipid peroxides [78]. This shift in redox balance promotes lipid peroxidation and sensitizes tumor cells to ferroptosis. Wang et al. first demonstrated that CD8^+^ T cell-derived IFN-γ contributes to the antitumor effect of immune checkpoint blockade by suppressing system x_c^−^ and enhancing ferroptosis in tumor cells [78]. In a subsequent mechanistic study, Yu et al. further showed that IFN-γ enhanced ferroptosis through JAK/STAT-mediated repression of SLC7A11, with chromatin immunoprecipitation supporting STAT1 binding to the SLC7A11 promoter [80]. In addition to restraining cystine metabolism, IFN-γ has also been linked to increased incorporation of polyunsaturated fatty acids into membrane phospholipids through ACSL4-associated remodeling, further increasing tumor susceptibility to ferroptotic damage [79]. Together, these findings indicate that CD8^+^ T cells can promote tumor cell killing not only through perforin/granzyme and Fas–FasL pathways, but also by imposing an IFN-γ-driven metabolic state that favors ferroptosis [78] (Figure 3).

### 4.3. Immunogenic Effects of Ferroptotic Tumor Cells

Ferroptotic tumor cells release immunogenic signals that influence antigen-presenting cells and promote adaptive immune responses [81]. During ferroptosis, tumor cells release damage-associated molecular patterns (DAMPs), including high-mobility group box 1 (HMGB1) and extracellular ATP [82]. These molecules function as danger signals that activate innate immune sensing pathways in the tumor microenvironment. Early ferroptotic tumor cells can therefore induce immunogenic cell death and promote dendritic cell activation [82]. Tang et al. demonstrated that HMGB1 and ATP released from ferroptotic cancer cells are critical mediators of this process and contribute to immune activation in vivo [81]. On the other hand, activated dendritic cells internalize tumor antigens derived from ferroptotic cells and undergo maturation, characterized by increased expression of antigen-presentation and co-stimulatory molecules such as MHC class II, CD80, and CD86 (Figure 3). Mature DCs subsequently migrate to lymphoid tissues, where they present tumor antigens to naïve T cells and initiate cytotoxic T-cell responses (Figure 3) [83].

This immunogenic potential is relevant to rectal cancer radiotherapy. Radiation can increase tumor antigen availability and inflammatory signaling, while ferroptosis may add a lipid-peroxidation-driven danger signal. If dendritic cells remain functional, these signals could support cross-presentation of tumor antigens and activation of cytotoxic T cells. In MSS/pMMR rectal cancer, where baseline immunogenicity is often limited, this mechanism could help explain why radiotherapy and ferroptosis modulation are being considered as partners for immunotherapy [83].

However, the timing and extent of ferroptosis are likely critical. Early or spatially restricted tumor-cell ferroptosis may provide immunogenic cues, whereas excessive or poorly controlled lipid peroxidation may damage immune cells and impair antitumor immunity. This distinction is important for therapeutic design. Ferroptosis should be induced preferentially in tumor cells and ideally during a window when dendritic cells and CD8^+^ T cells can respond productively.

### 4.4. The Immunosuppressive Side of Ferroptosis

Although ferroptosis can support antitumor immunity, its effects within the tumor microenvironment are not uniformly beneficial. Excessive lipid peroxidation can generate oxidized lipid species that impair antigen-presenting cells and weaken T-cell function. In dendritic cells, accumulation of oxidized lipids has been shown to suppress cross-presentation by reducing the ability of DCs to present tumor antigens through MHC class I, thereby limiting effective CD8^+^ T-cell priming. This is particularly relevant in MSS/pMMR rectal cancer [83], where baseline immune priming is often weak and successful immunotherapy likely depends on improving antigen presentation after radiotherapy [84].

Ferroptosis-related lipid stress can also directly compromise CD8^+^ T cells. In the tumor microenvironment, oxidized lipids may be taken up by CD8^+^ T cells through CD36, promoting lipid accumulation, T-cell dysfunction, and reduced antitumor cytokine production [85]. In addition, PD-1 signaling has been linked to altered phospholipid metabolism and increased susceptibility of intratumoral CD8^+^ T cells to ferroptotic death. Ping et al. showed that PD-1 signaling suppresses PLPP1 expression in CD8^+^ T cells through GATA1-dependent transcriptional regulation, thereby disrupting phospholipid homeostasis and promoting T-cell ferroptosis [14]. These findings suggest that ferroptosis can have opposite effects depending on the affected cell type. Ferroptosis in tumor cells may enhance tumor killing, whereas ferroptosis or lipid-peroxidation stress in dendritic cells and CD8^+^ T cells may suppress antitumor immunity [86]. Taken together, these studies indicate that ferroptosis has a dual immune role. Tumor-cell ferroptosis may enhance antitumor immunity, but ferroptotic stress in dendritic cells or CD8^+^ T cells may impair immune response. Therefore, future therapeutic strategies should aim for tumor-selective ferroptosis rather than generalized lipid peroxidation within the tumor microenvironment.

### 4.5. Radiation-Induced DNA Damage Activates cGAS–STING Signaling and Promotes Antitumor Immune Responses

Ionizing radiation induces DNA double-strand breaks and can promote the formation of micronuclei and cytosolic double-stranded DNA in tumor cells [87] (Figure 3). cGAS recognizes this cytosolic DNA and catalyzes the synthesis of the second messenger 2′3′-cGAMP. cGAMP then binds and activates STING at the endoplasmic reticulum, triggering its translocation to the Golgi apparatus, where STING recruits and activates TBK1 [87]. Activated TBK1 phosphorylates IRF3, leading to its nuclear translocation and induction of type I interferon and interferon-stimulated genes, including IFN-β [87,88] (Figure 3). These radiation-induced type I IFNs are critical for antitumor immunity. Deng et al. showed that cGAS-STING in dendritic cells is required for radiation-driven IFN-β production and CD8^+^ T cell priming and that exogenous cGAMP (mimicking this pathway) enhances radiation efficacy in vivo [89]. Downstream, type I IFNs act on myeloid and T cells. Lim et al. showed that type I IFNs (with IFN-γ) drive intraatumoral CXCL10 production after radiation, which correlates with CD8^+^ T cell infiltration, and directly enhances CD8^+^ T cell effector functions (IFN-γ and granzyme B production) [90]. Thus, radiation-induced cGAS–STING activation can convert irradiated tumors into immune-active sites. By generating new antigens and inflammatory cues, this pathway helps recruit and activate DCs and cytotoxic T cells, explaining the observed synergy between radiotherapy and checkpoint blockade [89,90]. For MSS/pMMR rectal cancer, this pathway provides a mechanistic rationale for combining radiotherapy with immune checkpoint blockade and ferroptosis-targeting strategies. Radiation may initiate DNA damage, cGAS–STING activation, type I IFN signaling, antigen release, and oxidative stress. Ferroptosis induction may amplify tumor-cell lipid peroxidation and death. Immune checkpoint blockade may preserve or restore CD8^+^ T-cell activity, including IFN-γ production, which can further suppress tumor-cell system Xc^−^ and increase ferroptotic pressure, as shown in Figure 3.

## 5. Targeting Ferroptosis to Overcome Radioresistance in Colorectal Cancer

### 5.1. SLC7A11-Mediated Antioxidant Defense in Colorectal Cancer

Colorectal tumors often upregulate antioxidant defenses that reduce ferroptosis and confer radioresistance [91,92]. When cystine/glutamate antiporter SLC7A11 (system Xc^−^) imports extracellular cystine in exchange for glutamate, fueling glutathione (GSH) synthesis and thereby sustaining GPX4-dependent lipid peroxide detoxification [93,94]. This redox system is frequently upregulated in CRC, with roughly half of patient tumors overexpressing SLC7A11 (TCGA data), and its expression correlates with advanced stage and therapy resistance [93,95]. By maintaining high intracellular cystine and GSH, SLC7A11 buffers radiation-induced ROS and protects membranes from peroxidation. In fact, ionizing radiation itself can trigger an adaptive antioxidant response. Tumor cells surviving RT often show elevated SLC7A11 and GPX4 expression [73,94]. Conversely, inhibition of SLC7A11 either by genetic knockdown or pharmacologic blockade with erastin rapidly depletes intracellular glutathione (GSH), leading to GPX4 inactivation and accumulation of iron-dependent lipid peroxides that drive ferroptosis. Multiple oncogenic and microenvironmental signals regulate this pathway. The tumor suppressor p53 normally represses SLC7A11 transcription and promotes ferroptotic vulnerability; however, factors such as CHI3L1 and the lncRNA FTX can counteract this effect and sustain SLC7A11 expression [96,97]. Mechanistically, FTX binds miR-625-5p and prevents it from repressing SLC7A11, leading to increased SLC7A11 expression [96]. Knocking down FTX in radioresistant CRC cells increases ROS and DNA damage under IR. Both of these effects can be reversed by SLC7A11 re-expression [94,96]. In parallel, the deubiquitinase USP5 stabilizes SLC7A11 through a YBX3-dependent mechanism; loss of USP5 promotes SLC7A11 degradation and enhances lipid ROS accumulation and ferroptosis following erastin treatment [98]. Together, these findings identify the SLC7A11–GSH axis as a central determinant of ferroptosis resistance and a targetable mechanism contributing to radioresistance in colorectal cancer.

### 5.2. GPX4 Dependence and Lipid Peroxide Detoxification

CRC cells, especially those with high polyunsaturated lipid content and labile iron, become critically dependent on GPX4 to prevent lethal lipid peroxidation. In preclinical models, direct GPX4 inhibition (e.g., by RSL3) induces cell death in CRC cells in a dose- and time-dependent manner [94]. RSL3 treatment sharply raises intracellular ROS and iron, inducing ferroptotic death that can be almost completely rescued by GPX4 overexpression or by ferroptosis inhibitors like ferrostatin-1 [94]. These observations demonstrate that GPX4 acts as a molecular shield against oxidative membrane damage. Similarly, colorectal cells that survive chemotherapy or chemoradiotherapy are characterized by elevated GPX4 (and accumulated Fe^2+^) to counteract oxidative stress [99]. These resistant tumor cells are exquisitely vulnerable to GPX4 inhibition, such as RSL3 or other GPX4 inhibitors that induce ferroptosis in drug-tolerant CRC cells [99]. Clinically, CRC biopsies after neoadjuvant chemoradiation show elevated GPX4 and ferritin levels, and high GPX4 expression predicts worse patient outcomes. Moreover, upstream regulators link GPX4 to radioresistance. For example, the NUAK1–Nrf2 pathway drives GPX4 transcription after irradiation. In LARC models, NUAK1 knockdown impaired Nrf2 nuclear translocation, reduced GPX4 upregulation, and markedly enhanced IR-induced ferroptosis [94]. Together, these data establish GPX4 as a critical ferroptosis defense in CRC and a key barrier to effective radiotherapy.

### 5.3. Ferroptosis-Inducing Drugs in Colorectal Cancer

Multiple small molecules can trigger ferroptosis in CRC cells. For example, erastin targets SLC7A11 to block cystine import, rapidly depleting GSH and disabling GPX4 [90,93]. In addition, erastin increases lipid peroxidation and induces ferroptotic death, an effect reversed by cystine supplementation or GPX4 re-expression [100]. Likewise, RSL3 covalently inhibits GPX4, leading to unchecked lipid-ROS accumulation. In HCT116, LoVo, and HT29 cells, RSL3 caused potent ferroptosis (via GPX4 loss) that is prevented by GPX4 overexpression [100]. Sorafenib, an FDA-approved multi-kinase inhibitor, also inhibits system Xc^−^. In CRC models, it elevates intracellular ROS and ferroptosis, especially when antioxidant capacity is compromised. Notably, combining sorafenib with radiotherapy shows synergistic effects. A phase II trial of sorafenib plus SBRT in CRC liver metastases overcame radioresistance in high-SLC7A11 tumors by inducing ferroptosis [94]. Other agents, such as sulfasalazine, statins, and artemisinin derivatives, have been reported to trigger ferroptosis via similar mechanisms. Experimental compounds FIN56 and FINO_2_ promote ferroptosis by driving GPX4 degradation and iron oxidation. Importantly, radiosensitization is a common theme among all these agents. Preclinical studies indicate that pairing radiotherapy with ferroptosis inducers converts sublethal IR doses into lethal oxidative damage in CRC [101,102]. Thus, disrupting SLC7A11–GSH–GPX4 defenses or enhancing iron-catalyzed lipid peroxidation leads to CRC cell death and can overcome radioresistance [103]. Table 1 exemplifies the translational landscape of ferroptosis-targeting strategies in colorectal cancer with representative FDA-approved drugs and experimental compounds, their proposed mechanisms, relevant CRC models, and key evidence supporting ferroptosis induction.

### 5.4. Proposed Combination Strategy: Radiotherapy, Ferroptosis Induction, and Immune Checkpoint Blockade in MSS/pMMR Rectal Cancer

**Figure 4:**
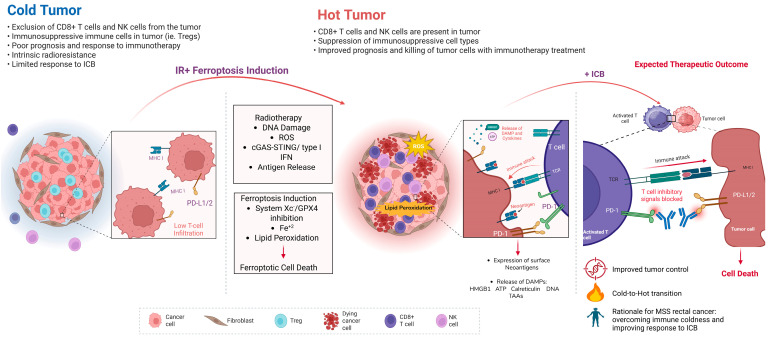
Proposed mechanism linking radiotherapy, ferroptosis induction, and immune checkpoint blockade in MSS/pMMR rectal cancer. Abbreviations: RT, radiotherapy; TNT, total neoadjuvant therapy; ICB, immune checkpoint blockade; DAMPs, damage-associated molecular patterns; TAA, tumor-associated antigen; TCR, T-cell receptor.

The proposed therapeutic model in Figure 4 integrates radiotherapy, ferroptosis induction, and immune checkpoint blockade as a rational strategy for MSS/pMMR rectal cancer. These tumors often show immune-cold features, including limited CD8^+^ T-cell and NK-cell infiltration, enrichment of immunosuppressive populations, and poor response to checkpoint blockade alone [110]. Radiotherapy may partially reverse this state by inducing DNA damage, reactive oxygen species, antigen release, and cGAS–STING/type I interferon signaling [111,112]. However, these effects may not be sufficient in tumors with strong antioxidant defenses or limited immune priming. Ferroptosis induction may enhance the therapeutic effect of radiotherapy by increasing iron-dependent lipid peroxidation and promoting tumor-cell death. Targeting the system Xc^−^–GSH–GPX4 axis, increasing Fe^2+^-driven oxidative stress, or enhancing PUFA-phospholipid peroxidation may lower the ferroptotic threshold of irradiated rectal cancer cells [27]. When tumor-cell ferroptosis is achieved, antigen release, HMGB1, ATP, cytokines, and other danger signals may promote dendritic-cell activation and T-cell priming, helping shift the tumor microenvironment from immune-cold to immune-hot [80,113].

Immune checkpoint blockade may further support this transition by restoring CD8^+^ T-cell effector function. Activated T cells can produce IFN-γ, which suppresses SLC7A11/SLC3A2 in tumor cells and weakens glutathione-dependent lipid peroxide detoxification, thereby reinforcing ferroptotic pressure [63,80]. Thus, radiotherapy, ferroptosis induction, and checkpoint blockade may act cooperatively. Radiotherapy initiates tumor inflammation; ferroptosis induction amplifies tumor-cell death, and checkpoint blockade sustains cytotoxic immune activity. Importantly, this strategy requires selectivity. Non-selective lipid peroxidation may impair dendritic-cell antigen presentation and promote CD36-mediated CD8^+^ T-cell dysfunction [85]. Therefore, the goal is not to induce broad ferroptosis across the tumor microenvironment, but selective ferroptosis in rectal cancer cells while preserving dendritic-cell and T-cell function. This concept provides a rationale for future biomarker-driven trials combining ferroptosis modulation with radiotherapy, TNT, and immunotherapy in MSS/pMMR rectal cancer.

## 6. Biological Challenges and Heterogeneity in Ferroptosis-Based Therapies

### 6.1. Metabolic Heterogeneity of Colorectal Tumors

Susceptibility to ferroptosis in colorectal cancer is determined by metabolic programs that regulate the availability of oxidizable membrane phospholipids and labile intracellular iron required for lipid-peroxide propagation. These programs vary across tumor subclones and directly influence how efficiently radiation-induced oxidative stress can be converted into ferroptotic cell death [85]. One major determinant is membrane lipid composition. ACSL4 activates long-chain polyunsaturated fatty acids (PUFAs), and LPCAT3 incorporates them into phosphatidylethanolamine, generating the phospholipid species most susceptible to iron-dependent peroxidation [95,96]. Tumor cells with high ACSL4–LPCAT3 activity therefore accumulate oxidizable PUFA-phospholipids and undergo stronger ferroptotic damage after radiation-induced ROS, whereas disruption of this axis lowers the pool of peroxidizable lipids and suppresses ferroptotic execution [114,115]. An opposing program is driven by lipid desaturation. SCD1 converts saturated fatty acids into monounsaturated fatty acids (MUFAs), which are incorporated into membrane phospholipids that are less permissive to lipid-radical propagation and restrict ACSL4-dependent PUFA enrichment. In colorectal cancer, oncogenic KRAS can reinforce this phenotype through SREBP1-dependent induction of FASN and SCD1, thereby promoting a MUFA-enriched, ferroptosis-resistant membrane state [115,116]. Inhibiting SCD1 reverses this shift, restores PUFA-phospholipid enrichment, and resensitizes tumor cells to oxidative lipid damage, making the KRAS–SREBP1–FASN/SCD1 axis a plausible target for ferroptosis-based radiosensitization [115,116]. Iron handling provides a second layer of heterogeneity. TfR1 upregulation increases iron import and expands the labile Fe^2+^ pool needed for Fenton-driven lipid oxidation, while NCOA4-mediated ferritinophagy releases stored iron and further amplifies peroxidation. By contrast, reduced ferritinophagy or increased ferroportin-dependent iron export lowers Fe^2+^ availability and restricts ferroptotic propagation [47,116]. Together, variation in phospholipid remodeling and iron metabolism establishes distinct ferroptotic thresholds across colorectal tumors. PUFA-rich, iron-loaded cells readily convert radiation-induced ROS into lethal membrane damage, whereas MUFA-enriched or iron-restricted cells remain relatively ferroptosis-resistant unless these metabolic barriers are therapeutically disrupted.

### 6.2. Tumor Microenvironment and Ferroptosis Regulation

Activated CD8^+^ T cells promote ferroptosis through IFNγ signaling, which suppresses SLC7A11 expression and limits glutathione-dependent lipid-peroxide detoxification while enhancing ACSL4-dependent incorporation of oxidizable PUFA-phospholipids [117]. This coordinated shift lowers the antioxidant threshold required to buffer radiation-induced lipid peroxidation and provides a mechanistic basis for synergy between ferroptosis induction and immune checkpoint blockade [67]. Myeloid cells further modulate ferroptotic susceptibility through local redox signaling. Macrophage-derived reactive oxygen species and inflammatory cytokines increase oxidative stress in adjacent tumor cells and amplify lipid-peroxide accumulation [118]. However, oxidized lipids within the tumor niche can impair dendritic-cell antigen presentation and promote CD8^+^ T-cell dysfunction through CD36-dependent uptake of peroxidized lipids, creating a feedback loop that limits effective antitumor immunity [85]. Hypoxia provides an additional regulatory layer by activating HIF-dependent transcriptional programs that increase lipid uptake and antioxidant buffering capacity, thereby restricting lipid-peroxide propagation despite elevated oxidative stress. In contrast, inflamed tumor regions favor cytokine-mediated suppression of cystine metabolism and promote ferroptotic execution [119]. Because radiotherapy simultaneously generates lipid-peroxidation-initiating ROS and enhances CD8^+^ T-cell recruitment, IFNγ-mediated repression of SLC7A11 represents a critical interface through which antitumor immunity amplifies radiation-induced ferroptotic tumor cell death [80,120].

### 6.3. Clinical Translation of Ferroptosis Targeting: Current Limitations and Trial-Level Evidence

Despite a strong preclinical rationale, clinical translation of ferroptosis-targeted therapy in colorectal and rectal cancer remains early. No ferroptosis-inducing strategy has yet demonstrated definitive clinical efficacy in rectal cancer, and most agents currently discussed in the clinical literature are indirect modulators of ferroptosis rather than selective ferroptosis-targeted drugs. This distinction is important because compounds such as system Xc^−^ inhibitors, iron-modulating agents, ROS-generating therapies, and artemisinin derivatives may influence ferroptosis-related biology without proving that ferroptosis is the dominant mechanism of clinical activity [78,121].

The most relevant CRC evidence comes from a small randomized preoperative pilot study of oral artesunate in patients undergoing CRC surgery, which suggested biological activity but was not powered to establish survival benefit or rectal cancer-specific efficacy [122,123]. The NeoART phase II trial further evaluates neoadjuvant artesunate in surgically resectable CRC; however, the study is currently suspended, and definitive efficacy outcomes have not been reported [NCT03093129] [124,125]. Importantly, NeoART does not directly test artesunate in the standard locally advanced rectal cancer setting of long-course chemoradiotherapy, short-course radiotherapy, total neoadjuvant therapy, or watch-and-wait management.

Sulfasalazine, an inhibitor of the cystine transporter system Xc^−^/SLC7A11, has also entered oncology trials as a ferroptosis-oriented strategy. Earlier studies in non-colorectal tumors, including recurrent malignant glioma and gastric cancer, showed limited clinical activity and raised feasibility or toxicity concerns [126]. In CRC, NCT06134388 is evaluating sulfasalazine in metastatic disease, but outcomes have not yet been reported, and the trial is not rectal cancer-specific and does not incorporate radiotherapy or immune checkpoint blockade [NCT06134388] [127]. Additional early-phase studies, including iron-loading nanomedicine approaches such as CNSI-Fe(II) [128], aim to increase labile iron and promote oxidative injury, but these studies remain exploratory and have not yet established clinical benefit in colorectal or rectal cancer [NCT06048367].

Taken together, current trial-level evidence supports ferroptosis as a plausible but still unvalidated therapeutic vulnerability in CRC. The major translational gap is the absence of biomarker-guided trials testing ferroptosis modulation in MSS/pMMR locally advanced rectal cancer, particularly in combination with radiotherapy or immunotherapy [110]. Current clinical trials exploring ferroptosis induction as an anticancer therapeutic strategy are summarized in Table 2. Future studies should integrate pharmacodynamic markers of ferroptosis, including SLC7A11, GPX4, ACSL4, FSP1, lipid peroxidation, and iron-handling markers, with immune profiling, ctDNA, clinical complete response, pathologic complete response, organ preservation, local regrowth, disease-free survival, and treatment-related toxicity [123].

## 7. Summary and Conclusions

Locally advanced rectal cancer is increasingly treated with multimodal strategies that combine radiotherapy, systemic chemotherapy, and, in selected molecular subgroups, immunotherapy [6]. Although these approaches have improved tumor downstaging and opened the possibility of non-operative organ preservation, treatment response remains highly variable [7]. Some tumors achieve durable complete clinical or pathological regression, whereas others persist despite similar radiotherapy and chemotherapy backbones. This heterogeneity indicates that radiation response is not determined by DNA damage alone, but also by tumor redox state, lipid metabolism, iron handling, antioxidant capacity, and immune contexture.

Ferroptosis provides a biologically coherent framework for understanding part of this variability. Ionizing radiation generates reactive oxygen species and lipid peroxidation, while ferroptosis defense pathways such as the SLC7A11–GSH–GPX4 axis, FSP1–CoQ10, DHODH, and GCH1–BH4 determine whether lipid oxidative stress remains controlled or progresses to lethal membrane damage [27,132]. In rectal cancer, this process must be interpreted within the actual treatment setting [12,27,37]. Radiotherapy is commonly combined with fluoropyrimidine-based chemotherapy, and modern total neoadjuvant therapy often includes oxaliplatin-based regimens or modified FOLFIRINOX. These treatments may alter ferroptotic thresholds through effects on nucleotide stress, mitochondrial function, redox adaptation, iron metabolism, and antioxidant buffering, although direct rectal cancer-specific evidence remains limited.

The immune consequences of ferroptosis are also context dependent. Tumor-cell ferroptosis may increase antigen release, danger signaling, and immune visibility, potentially supporting radiotherapy and immune checkpoint blockade strategies in MSS/pMMR rectal cancer [78,79]. However, ferroptosis should not be viewed as uniformly immunostimulatory. Oxidized lipids can impair dendritic-cell cross-presentation, and lipid uptake through CD36 can promote CD8^+^ T-cell dysfunction. Additional evidence suggests that extracellular GPX4 can impair dendritic-cell-mediated antitumor immunity through ZP3 receptor signaling. PD-1-linked phospholipid remodeling may further increase T-cell susceptibility to ferroptotic stress [78]. Therefore, the therapeutic goal is not broad lipid peroxidation across the tumor microenvironment but selective induction of ferroptosis in rectal cancer cells while preserving antigen presentation and cytotoxic T-cell function [89,90].

Clinically, ferroptosis-based strategies remain investigational. Existing trials and early translational studies provide important proof of concept, but none have yet established ferroptosis targeting as an effective treatment strategy for rectal cancer, nor have they defined how ferroptosis modulation should be integrated with long-course chemoradiotherapy, short-course radiotherapy, TNT, watch-and-wait management, or immunotherapy. Ongoing and future studies should therefore avoid treating ferroptosis as a generic anticancer mechanism and instead test it in clinically annotated rectal cancer cohorts with defined treatment schedules, paired tissue sampling, immune profiling, lipid peroxidation readouts, and organ-preservation endpoints.

In conclusion, ferroptosis sits at the intersection of radiation-induced oxidative stress, tumor metabolic vulnerability, and antitumor immunity. Its greatest translational potential in rectal cancer may lie in identifying tumors that are vulnerable or resistant to radiotherapy-based treatment and in designing rational combinations that enhance tumor-cell killing without compromising immune function. To move this field forward, future work must validate ferroptosis-related biomarkers in rectal cancer-specific cohorts, clarify how chemotherapy and radiation fractionation shape ferroptotic thresholds, and develop tumor-selective strategies that can be safely incorporated into TNT and organ-preservation approaches for MSS/pMMR rectal cancer.

## Figures and Tables

**Table 1:** Translationally relevant ferroptosis-inducing agents and supporting evidence in colorectal cancer models.

Agent	Reported Ferroptosis Mechanism	CRC Models (In Vitro/In Vivo)	Experimental Ferroptosis Evidence	Ref.
Sulfasalazine (FDA-approved; (ulcerative colitis)	System x_c^− inhibition	Radiosensitization of hypoxic CRC cells via GSH/TrxR depletion	C11-BODIPY lipid peroxidation; ROS assays; γ-H2AX DNA damage; rescue by ferrostatin-1; partial rescue by NAC; in vivo RT + SSZ schedule	[104]
Erastin	System xc^− inhibition	HCT116, LoVo, and HT29 CRC cells in vitro	Depletes cystine/GSH, disables GPX4; induces ferroptosis in CRC cell lines	[94]
Niraparib (PARP inhibitor; for ovarian and other indications) as ferroptosis amplifier with IR	Proposed: ↑IR-driven cytosolic dsDNA → cGAS–STING → ATF3–SLC7A11–GPX4 ferroptosis axis; plus, IFN-β–CD8 antitumor immunity	MC38, CT26, HT29 (CRC cell lines); mouse models; paired human rectal tumor samples pre/post RT (translational)	cGAS KD compromises IR-induced ferroptosis and CD8 infiltration; biomarkers include ATF3/PTGS2	[105]
Sorafenib (FDA-approved)	Xc^−^ inhibitor (plus kinases) and disrupt antioxidant defense, causing lipid peroxidation	DLD1(CRC cells), KM12-SM (mCRC cells) −/+ IR. Also, patient samples	Elevates ROS/ferroptosis in CRC; SBRT+sorafenib overcame mCRC radioresistance in a phase II trial	[103]
Oxaliplatin (standard CRC chemotherapy)	Oxidative stress + ferroptosis induction; studies propose NRF2 pathway involvement (context dependent)	HT29 and other CRC lines (study-dependent)	Ferroptosis/oxidative stress markers	[106]
Cisplatin (FDA-approved; not standard CRC backbone but commonly used cytotoxic)	Ferroptosis contribution to cytotoxicity (iron-dependent LPO)	HCT116 among tested tumor lines	Ferroptosis inhibitors and oxidative readouts reported in paper; HCT116 explicitly included	[107]
RSL3 (experimental GPX4 inhibitor; tool compound)	Direct GPX4 inhibition → ↑lipid peroxides	HCT116, LoVo, HT29 (in vitro CRC); MC-38 syngeneic CRC in vivo (as “RSL”)	In vitro: ROS/iron markers; GPX4 rescue reported; In vivo: improves anti-PD-1 efficacy	[108]
Ferric ammonium citrate (iron supplement; experimental iron-loading tool)	↑Iron availability may increase ROS/LPO; may sensitize to oxidative cell death while also inducing antioxidant adaptations	CRC in vitro (5-FU sensitization context)	FAC sensitizes CRC cells to 5-FU	[109]

**Table 2:** Current clinical trials exploring ferroptosis induction as an anticancer therapeutic strategy.

Trial ID	Title (Abbrev.)	Sponsor (Country)	Agents (Mechanism)	Combination	Indication	Phase	Enrollment	Status (as of 3/2026)	Primary Endpoints	Key Results/Status (Ref.)
NCT06134388	Sulfasalazine in metastatic colorectal cancer	Tanta Univ. Hosp (Egypt)	Sulfasalazine	System Xc^−^ inhibition; reduced cystine uptake and glutathione-dependent lipid peroxide detoxification	Metastatic colorectal cancer	III	50	Ongoing/no reported outcomes	Not reported	ongoing CRC-relevant clinical translation, not proof of efficacy
NCT06048367	CNSI-Fe(II) NP in advanced tumors	Sichuan Enray Pharma (China)	CNSI-Fe(II) nanoparticles (iron → ferroptosis)	Monotherapy	Advanced solid tumors (KRAS-mutant)	I	24	Active, recruiting	Safety (MTD, DLT)	Preliminary safety data pending [129]
NCT04205357	Sulfasalazine + SRS (CTNI-40)	Haukeland Univ. Hosp (Norway)	Sulfasalazine (system x_c^- inhibitor)	Stereotactic RT	Recurrent glioblastoma (GBM)	I	~12/cohort	Recruiting	Safety (MTD); PFS (explor.)	No results yet (protocol published) [130]
NCT03093129	NeoART-V: Artesunate vs placebo	108 Mil Central Hosp (Vietnam)	Artesunate (ROS generator; induces ferroptosis)	Placebo-controlled	Stage II/III colorectal cancer	II	200	Suspended (n = 200)	2-year PFS, OS	Trial suspended; interim safety OK [131]

## Data Availability

No new data were created or analyzed in this study.
